# The Burden of Hand Injuries at a Tertiary Hospital in Sub-Saharan Africa

**DOI:** 10.1155/2015/838572

**Published:** 2015-06-01

**Authors:** P. Makobore, M. Galukande, E. Kalanzi, S. C. Kijjambu

**Affiliations:** ^1^Department of Surgery, College of Health Sciences, Makerere University, Kampala, Uganda; ^2^Department of Surgery, Mulago National Referral Hospital, Kampala, Uganda

## Abstract

*Background*. Hand injuries are common worldwide and lead to heavy financial losses in terms of treatment, job loss, and time off duty. There is paucity of data on hand injuries in sub-Saharan Africa. The aim of this study was to determine the burden and early outcomes of hand injuries at a tertiary hospital. *Method*. A descriptive prospective study. Eligible patients were recruited over 5 months and followed up for four weeks. Pain, nerve function, and gross functions of the hand were assessed. *Results*. In total 138 patients were enrolled out of 2940 trauma patients. Of these, 122 patients returned for follow-up. The majority of the patients were males (83%). Mean age was 26.7 years (SD 12.8). The commonest places of injury occurrence were the workplace (36%), home (28%), and on the road (traffic crushes) (23%). Machines (21.3%) were the commonest agent of injuries; others were knives (10%) and broken glass (10%). Sixty-three (51%) patients still had pain at one month. *Conclusions*. Hand injuries accounted for 4.7% of all trauma patients. Road traffic crushes and machines were the commonest causes of hand injuries. Men in their 20s were mostly involved. Sensitization for prevention strategies at the workplace may be helpful.

## 1. Introduction

In this era of industrialization and reliance on machines, hand injuries are on the increase worldwide, accounting for 10–15 percent of admissions in emergency departments in the developed countries [1, 2]. Seventy percent of major hand injuries follow use of machines and are preventable [2].

In USA, 18 million hand injuries are seen per year [3]. In Uganda's largest hospital 6% of all trauma admissions in the emergency department are hand injuries.

Hand injuries contribute to financial losses in terms of treatment, time off work, and loss of job [4]. With increasing industrialization, hand injuries are on the rise and therefore increasing awareness and improving management of hand injuries are warranted in low resources settings.

Although much of the hand injuries are managed by plastic surgeons and orthopedic surgeons, hand emergencies are important in general practice [5]. In developing countries, they are usually managed by nonspecialists with little experience and training in management of hand injuries.

In one Ugandan study it was found that hand injuries were complicated by infections, gangrene, and loss of function. The infection rate was 63%. This study therefore set out to establish the pattern and early outcome of the hand injuries seen at Uganda National Referral Hospital in the capital, Kampala.

## 2. Methods

### 2.1. Design

A descriptive prospective study.

### 2.2. Setting

This study was carried out in the A/E Emergency Unit, Surgical Wards, and Surgical Outpatient Department of Mulago Hospital. The hospital is one of the main national referral hospitals and a teaching hospital for Makerere University Medical School. Being the largest and the oldest in the country, Mulago Hospital has a capacity of 1500 beds. The hospital receives about 20 000 surgical patients per year. About 50% of these are due to trauma. At least 30 patients with hand injury are seen every month, according to the records in A/E Unit, Mulago Hospital.

### 2.3. Selection Criteria

All patients with hand injuries seen, between October 2006 and February 2007, and who gave written informed consent were included. Patients below 18 years whose ascent was obtained were included.

Excluded were patients who presented with infected hand injuries and other associated injuries on the same limb like forearm fractures.

### 2.4. Data Collection and Analysis

Patients with hand injuries were interviewed, after triage by the doctor on duty in A/E Unit.

Age, sex, address, level of education, social class, and occupation of patients were recorded. Time, date, place of occurrence of hand injury, investigations done, and management plans were recorded using a questionnaire. Reviews at second and fourth weeks were conducted, and patients were assessed for the following: nerve function, pain, infection and gangrene, joint stiffness, and loss of digit(s). Gross function and healing were also assessed [6]. Severity of hand injury, using Tic-Tac-Toe method, was used to classify injuries into three types [7]: soft-tissue loss, bony loss, and combined tissue loss. Data is also presented as simple (no or minimal soft-tissue loss and complex (substantial soft tissues or/and body tissue loss).

Questionnaires were preread, corrected, and pretested.

Data were entered and analyzed using SPSS 11.5 statistical computer software. Continuous variables were analyzed using mean, median, range, and standard deviation. Categorical data was analyzed with proportions and frequency tables and summarized with frequency table and charts. Bivariate analysis of the factors affecting outcomes was done and presented as odd ratios and chi square.

### 2.5. Ethical Issues

Ethical approval was obtained from the Makerere University School of Medicine Institutional Review Board. Written informed consent was obtained from all participants.

## 3. Results

**Figure 1:**
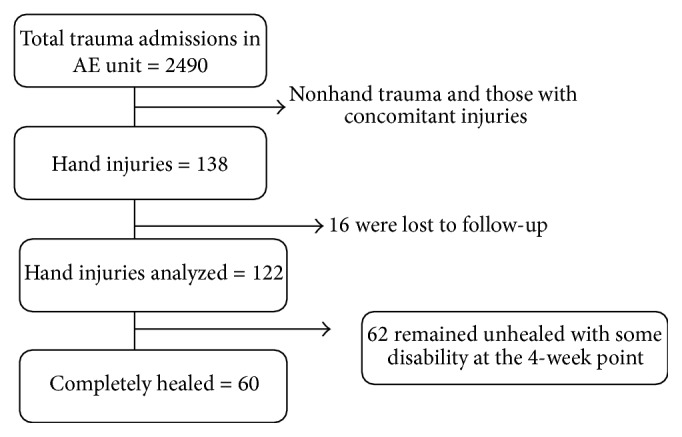
Flow chart patients seen in Accident and Emergency Department from October 2006 to February 2007 at Mulago National Referral Hospital.

During the study period, 2940 cases of trauma were seen in A & E Unit; of these a total of 138 patients were enrolled. Of these, 122 patients returned for follow-up at the two- and four-week point (see Figure 1). The percentage of hand injuries out of the total number of trauma patients was 4.7% (138/2940).

The mean age was 26.7 (SD12.8). There were more males injured with the ratio M : F 5 : 1. Most (95%) patients were right handed and 68% (94/138) were in informal employment (see Table 1).

After a follow-up period of 4 weeks, 80% of patients still had pain, 27% had infection, 7.4% had at least one stiff joint, 14.8% had lost part of or finger(s), and 43–53 had lost gross functions including pinch and grasp. Only 45% had completely healed and were free of symptoms (see Table 2).

In Table 3, the nature of injury and agent are shown as simple and complex and crush is shown as a more severe form of injury. Home kitchen knife and glass (bottle and window) related injuries were likely to be complex in nature (*P* < 0.010). More severe (crush) injuries were likely to occur at workplaces (borderline significance *P* = 0.080) and injuries occurring at home were likely to be less severe (simple) *P* < 0.001.

Occupation had no influence on the severity of injury save for the skilled category OR 2.30 *P* = 0.020.

## 4. Discussion

We set out to investigate the burden, causes, and short outcomes of hand injuries in patients presenting at Uganda's largest urban hospital in Kampala. We found that most injuries occurring on the road and at work were caused by road traffic crushes and machines, respectively. Young males were mostly involved and half (50%) of them were unable to fully use the hand (mostly the dominant ones) after 4 weeks. The male to female ratio was 4 : 1, similar to other studies [5, 8]. Though we did not quantify loss, half of the participants were unable to fully use the injured hand for work for at least a month. We know that a slight injury to a finger or to the hand may rob a young man of his livelihood and alter the whole course of his life [9]. The injured people in the work place were mostly semi- or unskilled workers whose livelihood depends on the manual work, for example, farming and crafts workers, on a daily basis to earn a daily wage [10–12].

The reasons for machine injuries were not explored nor were the types of machines; probably injuries were due to a lack of training before using these machines or/and lack of protective wear which may contribute to occurrence and severity of hand injuries. Injuries due to knives were probably due to careless use and glass and due to accidental trauma. Trybus and associate reported that 34% of their patients were injured by mechanical equipment [13].

Assault (violence) and self-affliction accounted for 23% of the hand injuries. Many factors account for the violence, one of which is increased urbanization and high levels of unemployment [14]. Road traffic crushes were a significant contributor of traumatic hand injuries as was reported 18 years ago at the same study site; the situation seems not to have changed.

Injuries occurring at home were likely to be less severe (simple) as compared to injuries occurring at the work place. This relates to the agent of injury. The skilled category was likely to suffer the more severe injuries perhaps this has to do with the nature of machinery used.

Females sustained most of the hand injuries at home, doing domestic chores. Females with limited education are likely to become housemaids, doing home chores [15].

Unlu reported that 70% of his patients were male [5] while Momcilovic and Brokes reported a male preponderance (85%) in their series [8].

The average age of the injured in this study was 26 years. This falls in the most active age group. They usually have little experience in their vocation and hence are prone to hand injuries. This does not differ from other studies. Katerega found that the average age was 30 years [9]. Sahin et al. reported 28 years [15]. However, the average age in North America is 37 years [13].

In this study, ninety-five percent of the patients were right handed. This concurs with the proportion of the right handed people in the general population [16]. However, the left hand was slightly more injured than the right hand (51% and 45% resp.). This is because hands play different roles in everyday activities. Only a small number (4%) had both hands injured. This can be explained by the fact that usually the left hand holds the object while the right hand cuts making the left hand prone to injuries; however other studies are different [2, 11]. The difference could be due to variations in the occupations. For the parts injured, the palm zone 3 and the dorsum of the hand were almost equally injured, 36% and 32%, respectively, in this study. In 32% both aspects of the hand were injured. The dorsum of the hand is prone to injuries because of thin skin and weak reflexes of the extensors [17]. Strong palm fascia and the quick reflexes of the flexors of the hand protect the palm.

All together, the work places, homes, and crushes on the road were responsible for 90% of all the hand injuries. At homes careless handling of knives may probably explain the high incidence of hand injuries. There is also the increased use of machines in urban homes like mowers [18]. Road traffic crushes are a significant cause of morbidity and mortality in Uganda, and the hand is not spared. A slightly different picture was found in Hong Kong where 65% cases occurred at work, 15% at home, and 6.5% were due to road traffic accidents [11, 14].

We found that half of the patients injured still had pain after one month. Hand injuries are painful, because the hand is richly supplied with somatic nerves [17]. Infection was the next commonest complication after pain. The lost fingers and stiff joints all of these contributed to capacity and disuse. On average, hand injuries require about six weeks to heal [13]. Trybus and coworkers estimated average time of healing to be 77 days. They also noted that 58% of their patients had permanent impairment. They concluded that treatment of complex hand injuries requires specialized centers, and these centers and hand surgeons are limited or nonexistent in many low income countries [13].

Patients with crush injuries were likely to take long time to heal than those in simple injuries (OR = 0.03, 5.30). The odds ratio for healing of fractures compared to other injuries was 13.0. Fractures take from four to six months to heal [19].

In the management of hand injuries, an X-ray is the most important single investigation, especially in crush injuries, which are associated with fractures and/or dislocations. Other investigations like ultrasound (include Doppler) and MRI are important [20], but these are not readily available in low income countries.

Surgical toilet and suture, debridement, splintage, and tendon repairs were the procedures done. These procedures may not require a major operating theatre, but they must be done well. The consequences of the injury can be reduced by proper assessment, appropriate treatment and careful follow-up, but better prevented [12].

Nonoperative management included elevation, dressing, antibiotics, analgesia, and physiotherapy. Elevation minimizes swelling and pain. Antibiotics treat or prevent infection. Analgesia controls pain and physiotherapy aids early return to full function.

Long distances and time delays to the hospital may impact outcomes negatively [21]. In this study the average time from injury to presentation was ten hours, and the range was from 30 minutes to 100 hours. This delay could be explained by a lack of a functional ambulance system and a broken down referral system. Excessive delay predisposes to establishment of infection and blood loss.

The odds ratio for time delay of less than six hours and 6 to 12 hours was 0.19 and 2.83, respectively.

*Limitations*. A follow-up period of four weeks may have led to overestimation of bad outcomes, because some injuries like tendon injuries and fractures normally need up to six months or more to completely heal. Sixteen patients were lost to follow-up, and therefore we had no opportunity to assess recovery.

## 5. Conclusion

The burden of hand injuries was 5.5% of all the trauma patients seen. The degree of incapacity was significant as fifty percent of the participants were unable to use the injured hand at the end of one month. The commonest place and cause of hand injuries are work place and machines, respectively. Targeted campaigns to sensitize workplaces and reduce on the road carnage may contribute to prevention of hand injuries.

## Figures and Tables

**Table 1:** Showing study participant characteristics.

Characteristic	Frequency	Percentage
Gender		
Male	115	83
Female	23	17
Education		
Primary	56	40
Secondary	46	33
Tertiary	21	27
Handedness		
Right handed	131	95
Left handed	7	5
Occupation		
Formal	44	32
Nonformal	94	68
Distance from hospital		
<5 km	67	49
5–10 km	49	36
>10 km	22	15
Place of injury		
Work	50	36
Home	40	29
Road (traffic injuries)	34	25
Farm/garden	4	3
Sports	1	0.5
Others	9	6.5
Types of injuries		
Lacerations	39	32
Crush	29	25
Fractures	18	16
Tendon injuries	8	7
Others^*∞*^	44	20
Operative treatment		
Surgical toilet & suture	50	36
Debridement	50	36
Splintage	25	18
Tendon repair	8	6
Time of surgery (after arrival)		
Less than 6 hours	94	68
6–12 hours	27	20
12–24 hours	6	5
Above 24	3	2
Missing	6	5

The 9 patients that had not healed after 4 weeks had presented >12 hours after injury. None of the healed ones had presented ≥12 hours after injury.

^*∞*^Others included a mix of injuries from say bites, abrasions, confusions, and bruising.

**Table 2:** The table shows postinjury outcomes after 4 weeks of follow-up.

Outcome (*N* = 122)	Frequency	Percent (%)
Pain	98	80
Infection	35	27
Gangrene	2	1.7
Stiff joints	9	7.4

Loss of fingers (amputation)	18	14.8
Gross function		
Pinch	73	53
Grasp	59	43
Opposition	72	52
Healed	54	45

**Table 3:** The table shows time of surgery healing and type of injury.

	Healing		OR	*P* value	C.I
	Nature of injury				
	Simple	Complex			
Agent					
Knives^*∗*^	13	1	0.09	0.010	0.00–0.72
Machine	7	7	1.63	0.390	0.47–5.57
Glass^†^	11	18	2.92	0.010	1.15–7.49
Burns^*∗∗*^	4	0	0.00	0.090	0.00–2.16
Others	43	29	—	—	—
Place of occurrence					
Home	30	7	0.19	<0.001	0.07–0.52
Work	0	27	0.79	0.080	0.87–4.1
RTC	23	15	1.90	0.748	0.47–2.75
Sports	17	1	1.14	0.820	0.08–8.22
Occupation					
Business	7	8	1.73	0.320	0.52–5.74
Unskilled	18	12	0.93	0.860	0.37–2.30
Skilled	21	25	2.30	0.020	1.04–5.10
Students	7	4	0.80	0.720	0.18–3.25
Professionals	4	3	1.07	0.930	0.18–5.97

C.I: confidence interval.

^*∗*^Home kitchen knives.

^†^Glass: bottle and window glasses.

^*∗∗*^This miscellaneous section included agents such as animal and human bites, slammed doors, nail piercing, falls, and panga (machete) cuts.
